# Blood and Dried Blood Spot Telomere Length Measurement by qPCR: Assay Considerations

**DOI:** 10.1371/journal.pone.0057787

**Published:** 2013-02-25

**Authors:** DeAnna L. Zanet, Sara Saberi, Laura Oliveira, Beheroze Sattha, Izabella Gadawski, Hélène C. F. Côté

**Affiliations:** Department of Pathology and Laboratory Medicine, University of British Columbia, Vancouver, British Columbia, Canada; Instituto Butantan, Laboratório Especial de Toxinologia Aplicada, Brazil

## Abstract

Measurement of telomere length is crucial for the study of telomere maintenance and its role in molecular pathophysiology of diseases and in aging. Several methods are used to measure telomere length, the choice of which usually depends on the type and size of sample to be assayed, as well as cost and throughput considerations. The goal of this study was to investigate the factors that may influence the reliability of qPCR-based relative telomere length measurements in whole blood. Day to day intra-individual variability, types of blood anticoagulant, sample storage conditions, processing and site of blood draw were investigated. Two qPCR-based methods to measure telomere length (monoplex vs. multiplex) were also investigated and showed a strong correlation between them. Freezing and thawing of the blood and storage of the blood at 4°C for up to 4 days did not affect telomere length values. Telomere lengths in dried blood spots were significantly higher than both whole blood and peripheral mononuclear blood cells, and were highly correlated with both. We found that telomere length measurements were significantly higher in dried blood spots collected directly from fingertip prick compared to dried blood spots prepared with anticoagulated whole blood collected from the finger, and non-blotted whole blood taken from both finger and arm venipuncture. This suggests that DNA from cells blotted on paper is not equivalent to that collected from venipuncture whole blood, and caution should be taken when comparing between blood sample types.

## Introduction

Telomeres are DNA-protein structures found at the end of linear eukaryotic chromosomes [1]. In humans, telomeric DNA consists of a highly conserved repetitive DNA sequence (TTAGGG)_n_, approximately 10–15 Kb in length, that does not code for proteins, but is associated with telomere-binding proteins and is of critical importance to cells [2], [3]. Telomere functions include regulation of cellular replicative capacity, as well as stabilization and protection of chromosomal ends [4]. Telomere length (TL) shortening has been associated with aging, stress, increased risk for many age-associated conditions and diseases [5], [6]. In particular, leukocyte telomere length (LTL) has been reported to predict mortality and cardiovascular events [7]. Consequently, there is great interest in measuring TL in various tissues, most commonly in blood.

Several methods exist to measure TL [8]. Among the most established and widely used, is telomere restriction fragment (TRF) analysis. However, measurements include a region of subtelomeric DNA that can vary in length between individuals, potentially confounding inter-individual comparisons [8], [9]. Flow-fluorescence *in situ* hybridization (flow-FISH)is another method used to measure the average length of telomere repeats in sub-populations of blood cells[9] but it is not practical for use with solid tissues or archived samples. TRF and flow-FISH typically require a minimum sample of approximately 1 µg of DNA or ∼1–2 mL of fresh blood. Furthermore, both assays are relatively labor-intensive and time-consuming, something that may limit their applicability for large-scale studies [10]. Cawthon developed a rapid and inexpensive qPCR-based TL assay using a partial mismatch primer strategy [11]. This method quantifies fluorescence signal proportional to average telomere length (T) in a sample, relative to a single nuclear gene copy number (S). This enables the use of small and/or archived samples, and high throughput. As of March 2012, more than 300 publications to date have used this qPCR method, as per Thomson Reuters’ Web of Knowledge. More recently, a dot blot-based method was developed [12] that reportedly requires little DNA and shows a high reproducibility but it has not yet been widely used in the field.

Large-scale epidemiological studies of human TL frequently use banked tissue or DNA samples originating from multiple sites. Variations in the procedures used to collect, process, and store clinical samples are likely encountered in this context. Venipuncture, a common method to obtain blood is relatively laborious, invasive, and can be expensive for large-scale studies [13] such that in resource-poor or remote areas, venipuncture may not be practical. Blood collected directly from fingertip puncture onto paper as a dried blood spot (DBS) is considered stable at room temperature and is often favored by many investigators [13].

Accurate reliable measurement of TL is critical to research on the biological and clinical significance of telomeres. The goal of our study was to investigate the robustness of TL measurement by qPCR in blood, and the various factors that may influence its measurement.

## Materials and Methods

### Study sample collection

**Figure 1:**
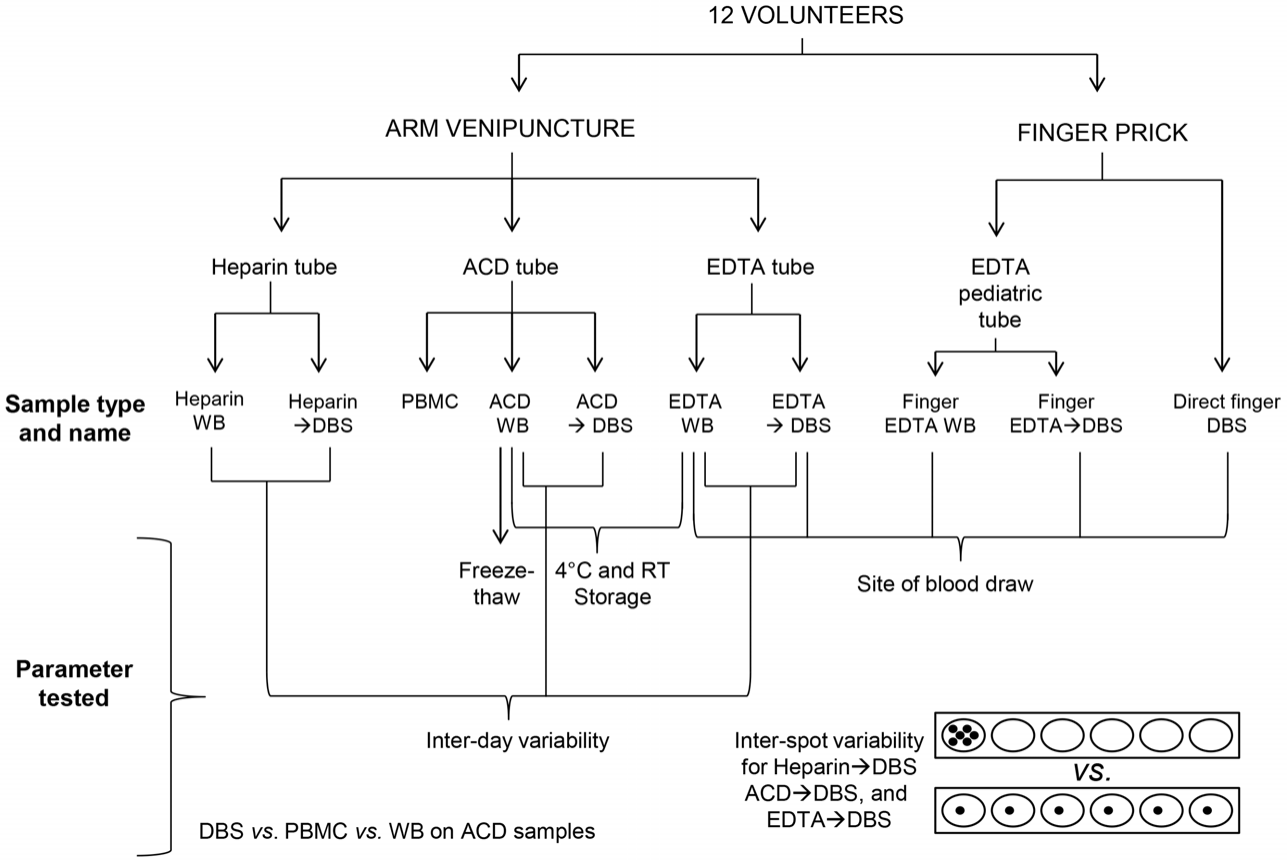
Sample collection overview. Diagram outlining the sites of blood sample collection, anticoagulants used, sample processing, and parameters tested for each sample type.

Peripheral whole blood (WB) samples were collected by arm venipuncture (BD vacutainers containing ethylenediaminetetra acetic acid (EDTA), acid citrate dextrose (ACD) solution A or heparin as anticoagulant) or by finger prick using BD Microtainer Contact Activated Lancet (30 G×1.5 mm). Arm venipuncture WB was either spotted onto Whatman903 Multipart Neonatal cards (GE Healthcare) to prepare dried blood spots (DBS) consisting of six spots of 80 µL each), frozen without processing, or used to prepare peripheral blood mononucleated cell (PBMC). PBMCs were isolated from 3 mL of fresh ACD blood by Ficoll-Paque PLUS density gradient centrifugation (VWR). The cells were resuspended in 1 mL ice-cold serum-free medium with 10% DMSO, slowly frozen and stored at -80°C until DNA was extracted the next day. Finger prick blood was either collected into pediatric tubes (BD Microtainer with EDTA), or blotted directly onto paper to prepare DBS. All samples, unless otherwise indicated, where stored at -80°C until DNA extraction. Figure 1 shows a diagram of the various study samples collected and the experiments carried out.

### DNA Extraction

All DNA extractions were performed using the QIAcube and QIAamp DNA Mini Kit (Qiagen) according to the manufacturer’s protocol (www.qiagen.com/literature/render.aspx?id=200373).WB total DNA was extracted using the Blood and Body Fluid Protocol with the following modifications : 100 µL of WB, diluted with 100 µL of 1x PBS, was used and the DNA was eluted in 100 µL buffer AE. Samples of the same type (*e.g.* all ACD-containing WB) were extracted in batch.

DBS total DNA was extracted using the Dried Blood Spot protocol with the following modifications: for each DBS, DNA was extracted from six 3 mm diameter paper punch discs obtained from a single spot. For the automated QIACube extraction protocol, 350 µL of buffer ATL, 20 µL of Proteinase K solution, 300 µL of buffer AL and 150 µL of 95% ethanol were used. The DNA was eluted in 50 µL of buffer AE. In addition, to assess variability between adjacent spots on one DBS specimen collection paper, a separate extraction procedure was performed in which the punched-out discs were obtained from 6 different spots containing the dried blood, as described above. Again, the samples of similar type were batched.

PBMC samples were thawed, centrifuged at 8000 x *g* for 10 min and the cells resuspended in 200 µL 1X PBS. Total DNA was extracted using the Blood and Body Fluid Protocol with a final elution volume of 100 µL in buffer AE.

### Quantitative PCR

For most results presented herein, quantitative real-time PCR (qPCR) amplification was performed as described [11], [14] using LightCycler® 480 (Roche) with the following modifications. The accessory subunit of polymerase gamma (ASPG or POLG2) was used as the single copy nuclear gene for (S) copy number determination with primers ASPG3F (5′ GAG CTG TTG ACG GAA AGG AG 3′) and ASPG4R(5′ CAG AAG AGA ATC CCG GCT AAG 3′) at 1 µM final concentration each. For the quantification of telomere (T), tel 1b (5′ CGG TTT GTT TGG GTT TGG GTT TGG GTT TGG GTT TGG GTT 3′) and tel 2b (5′ GGC TTG CCT TAC CCT TAC CCT TAC CCT TAC CCT TAC CCT 3′) were used at 0.3 and 0.9 µM, respectively. For each PCR reaction, 8 µL of telomere (T) or ASPG (S) master mix and 2 µL of DNA were added to wells of a96-well plate (Roche), in duplicate. The LightCycler® 480 SYBR Green I Master (Roche) was used for both T and S qPCRs. A standard curve was included in each run and prepared by serial dilutions (1∶2) of pooled human WB DNA, yielding DNA ranging from 30,000 to 469 copies of S and 90 to 1.41 relative copies of T corresponding to total DNA concentrations of ∼13.8 ng/µL to 0.22 ng/µL. Prior to PCR, the plates were centrifuged at 1500 x *g* for 2 min at room temperature. The thermal cycling for both PCR reactions began with a 95°C incubation for 10 min. For S PCR, this was followed by 45 cycles of 95°C for 5 s, 60°C for 10 s, and 72°C for 5 s. For T PCR, this was followed by 45 cycles of 95°C for 5 s, 54°C for 30 s, and 72°C for 1 min. The ramping temperature rate to the annealing step was set at 2.2°C/s for S and 1.0°C/s for T unless otherwise indicated. Absolute quantification results for both T and S were obtained using the Second Derivative Maximum method with the LightCycler® 480Software v. 1.5.The relative telomere length value was expressed as the average of duplicate T*1000 over the average of duplicate S (T/S). We previously showed an excellent correlation between telomere length measurements done by monoplex qPCR and flow-FISH [14].

A newer multiplex qPCR telomere assay was published shortly after this study was carried out[15]. To ascertain how the two methods compared, a set of 32 WB samples collected from subjects aged between 2 and 59 years were assayed with both the two-tube monoplex assay described above [11] and the newer single-tube multiplex assay [15] using albumin as the single copy gene (S). Both assays were performed on a LightCycler 480. Briefly, each 10 µL reaction mix contained 2 µL of sample DNA (∼20 ng/µL) and 8 µL of master mix. The final concentrations of reagents in the master mix were 1X LightCycler® 480 SYBR Green I Master (Roche) and 0.9 µM of each of the following four primers: albu (5′ CGG CGG CGG GCG GCG CGG GCT GGG CGG AAA TGC TGC ACA GAA TCC TTG 3′, albd (5′ GCC CGG CCC GCC GCG CCC GTC CCG CCG GAA AAG CAT GGT CGC CTG TT 3′), telg (5′ ACA CTA AGG TTT GGG TTT GGG TTT GGG TTT GGG TTA GTG T 3′), and telc (5′ TGT TAG GTA TCC CTA TCC CTA TCC CTA TCC CTA TCC CTA ACA 3′). The thermal cycling profile was 95°C for 15 min, followed by 2 cycles of 94°C for 15 s,49°C for 15 s, followed by 40 cycles of 94°C for 15 s, 62°C for 10 s, 74°C for 15 s, 84°C for 10 s, and 88°C for 15 s, with signal acquisition at the end of both the 74°C and 88°C steps. A standard curve was generated by serial dilutions (1:2) of pooled human buffy coat DNA ranging from 224,000 to 875 copies of albumin (S) and 928 to 4 relative copies of T. This corresponds to a total DNA concentration of ∼130 to 0.51 ng/µL. Measurements were carried out in duplicate and the LightCycler raw text files were converted to grid format using LC480Conversion free software developed by the Heart Failure Research Center (HFRC) in Amsterdam, the Netherlands (http://www.hartfaalcentrum.nl/index.php?main=files&fileName=LC480Conversion.zip&description=LC480%20Conversion&sub=LC480Conversion). The converted data were analyzed using LinRegPCR free software developed by Rutjer *et al.* [16]. T and S copy numbers were calculated based on the standard curves. As for the monoplex assay, the relative telomere length value was expressed as the average of duplicate T*1000 over the average of duplicate S (T/S).

#### qPCR assay Quality Control

For both assays, duplicates with absolute difference >20% were rejected and repeated. Three positive internal controls (IC) were included in each run (a high, a medium and a low T/S) and their inter-run coefficient of variation (CV: SD*100/mean) was monitored. For each run, PCR efficiency, standard curve errors and IC values were monitored and had to lie within mean±2 SD for Quality Control.

### Statistics

Comparisons between groups were done using Mann-Whitney, Wilcoxon signed-rank, Friedman’s or t-test, as appropriate. Pearson’s correlations were also calculated. Statistical analyses were done with χLSTAT 2009 software version 4.06. P ≤ 0.05 (double-tailed) was considered statistically significant.

### Ethics statement

The study was approved by the University of British Columbia Research Ethics Board (H08-01506). All volunteer blood donors provided written informed consent.For younger donors, written informed consent was provided by a parent or guardian.

## Results

### qPCR assay conditions

#### Variability

**Figure 2:**
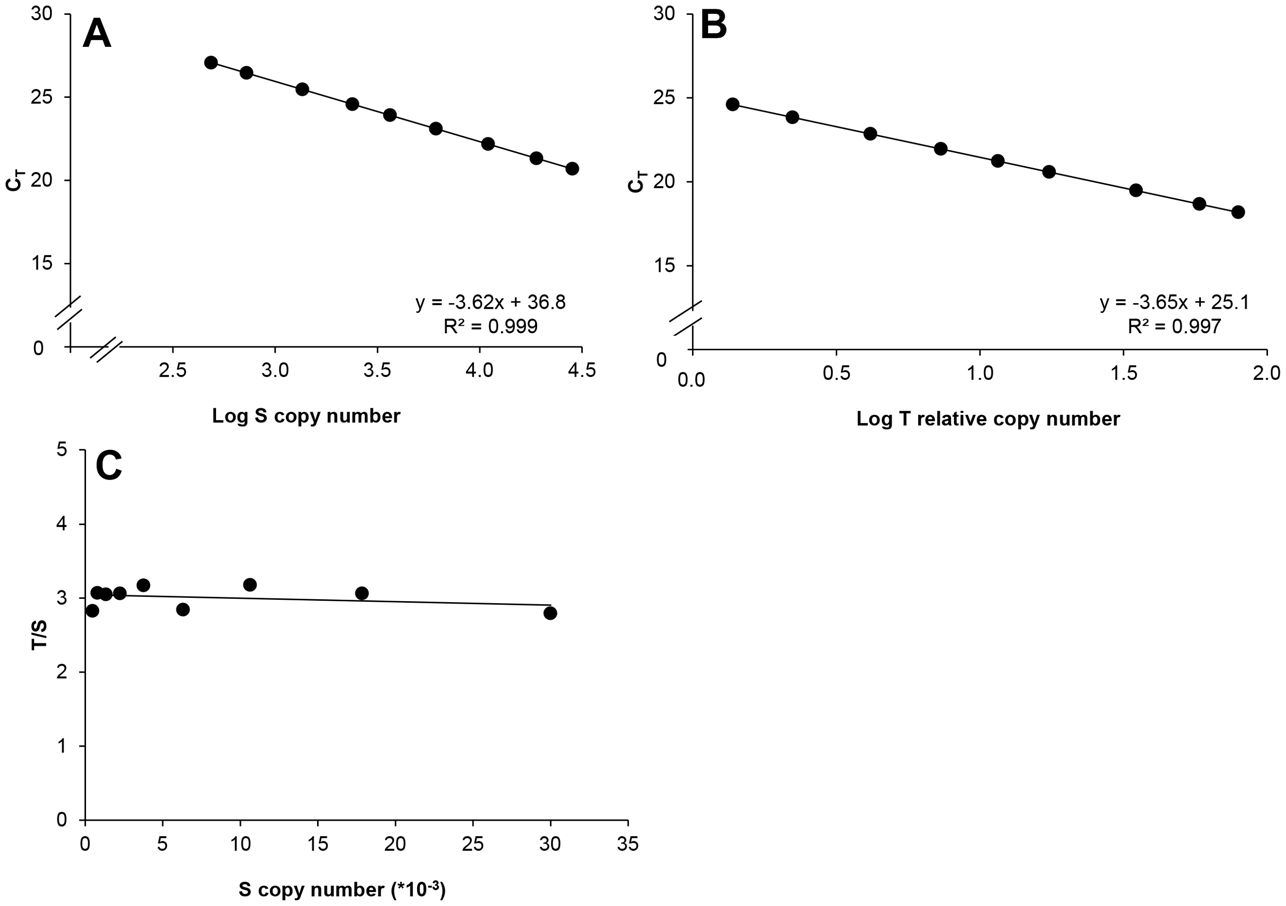
Standard curves used to determine T/S ratio. Pooled blood DNA was serially diluted (1:2) to generate nine DNA standards for the ASPG accessory gene (S) (panel A) and the telomere region (T) (panel B). Stability of the T/S ratio within the range of DNA concentrations was tested (panel C). N = 1 for each point.

Although T/S should theoretically be independent of DNA concentration, in practice, it can diverge from linearity at extreme concentrations. To determine the stable range of the assay, pooled blood DNA was serially diluted (1:2) and both T and S were assayed. Figure 2 (A,B) show standard curves for T and S, while Figure2C illustrates the stability of T/S over the DNA concentration range studied. The PCR efficiencies were similar for both and T/S was stable over the whole range of DNA concentrations tested. To determine the assay’s variability, the intra-assay (n = 10) and inter-assay (n = 38) CV for S, T and T/S values were calculated for three IC (Table 1). In general, the medium range IC showed the highest intra- and inter-assay CV for S and T copy number (5.8–16.7%)and C_T_(0.5–2.1%) while IC low tended to show the lowest CV. As expected, C_T_ variability was lower than that of absolute copy numbers derived from the standard curves.

**Table 1:** Intra-assay and inter-assay variability for the calculated copy number and cycle threshold (C_T_) for the three internal controls (IC).

	Intra-assay (n = 10)						Inter-assay (n = 38)				
	Copy number [Mean ± SD (%CV)]										
	S		T		T/S		S	T		T/S	
IC high	8032 ± 512 (6.4)		36.1 ± 2.5 (7.0)		4.5 ± 0.4 (8.9)		7490 ± 453 (6.0)	36.5 ± 3.0 (8.2)		4.9 ± 0.4 (9.6)	
IC med	2939 ± 250 (8.5)		10.5 ± 0.6 (5.8)		3.6 ± 0.4 (11.4)		3082 ± 275 (8.9)	9.4 ± 1.4 (15.5)		3.0 ± 0.5 (16.7)	
IC low	1899 ± 62 (3.2)		5.8 ± 0.2 (3.7)		3.1 ± 0.2 (5.0)		1903 ± 73 (3.8)	5.6 ± 0.2 (4.3)		3.0 ± 0.2 (6.4)	
	C_T_ [Mean ± SD (%CV)]										
	S	T		T/S		S			T		T/S
IC high	23.8 ± 0.1 (0.4)	14.1 ± 0.1 (0.8)		---		23.9 ± 0.1 (0.3)			14.5 ± 0.3 (1.8)		---
IC med	25.3 ± 0.1 (0.5)	16.0 ± 0.1 (0.6)		---		25.2 ± 0.3 (1.1)			16.9 ± 0.4 (2.1)		---
IC low	26.0 ± 0.05 (0.2)	16.9 ± 0.1 (0.4)		---		26.0 ± 0.1 (0.4)			17.7 ± 0.3 (1.7)		---

Abbreviations: SD  =  standard deviation, CV  =  coefficient of variation, S  =  single copy gene, T  =  telomere repeats, T/S  =  relative telomere length ratio, IC  =  internal control, C_T_  =  cycle threshold

#### Concordance between relative telomere length measured using monoplex and multiplex qPCR

**Figure 3:**
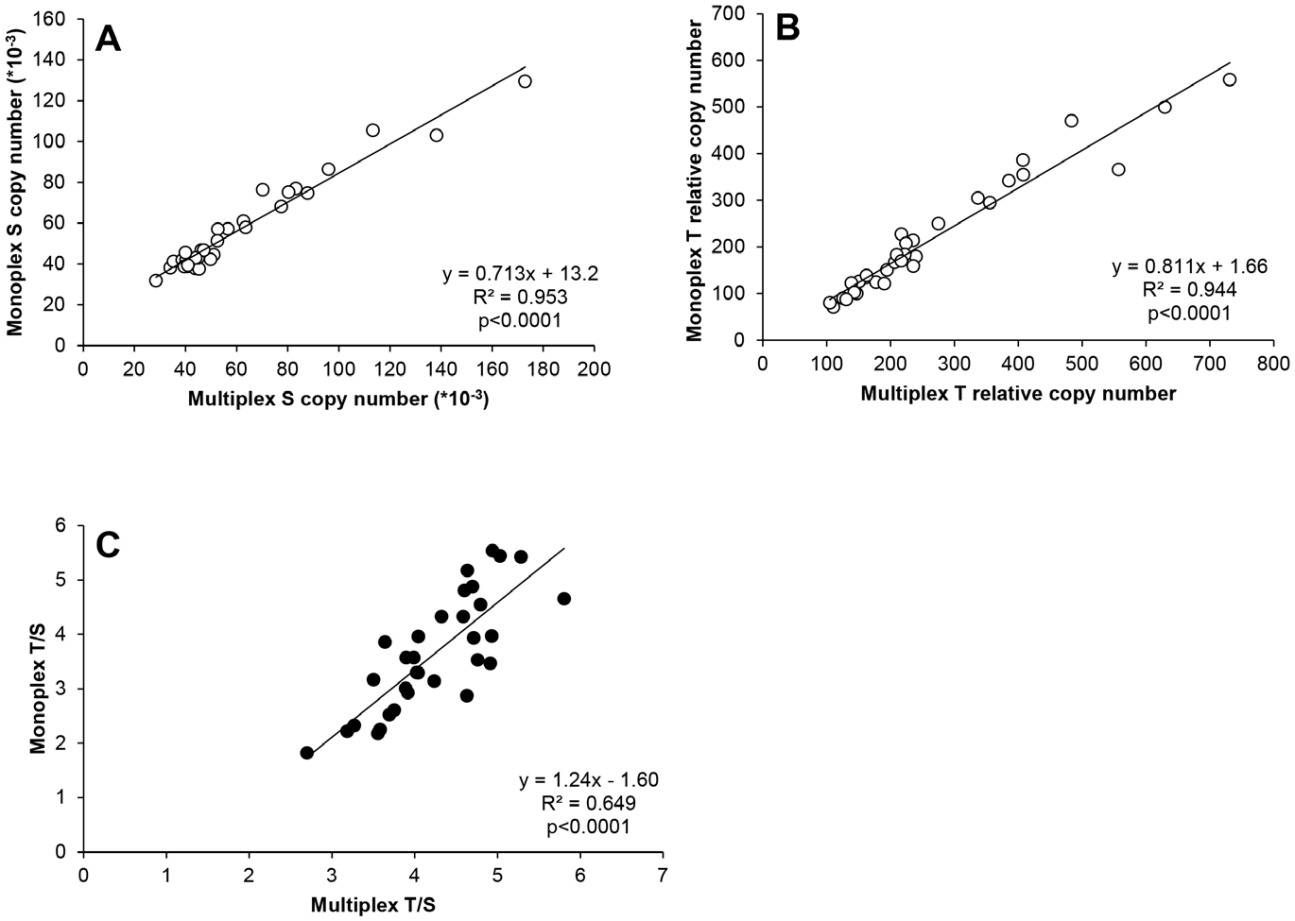
Correlation between monoplex and multiplex qPCR. ACD WB (N = 32) samples were measured by monoplex and multiplex qPCR. Correlations were done between S (panel A), T (panel B) and T/S (panel C). Pearson’s R**^2^** and p values are shown.

Multiplex qPCR was performed on WB samples spanning a range of T/S ratios. A strong correlation was observed for each individual gene (S, R^2^ = 0.953, p<0.0001; T, R^2^ = 0.944, p<0.0001) measured by the two methods (Figure 3 A,B), although that for the T/S ratio (R^2^ = 0.649, p<0.0001, Figure 3 C) was less strong, as could be expected.

### Relative telomere length measurements in blood

#### Effect of blood anticoagulant

**Figure 4:**
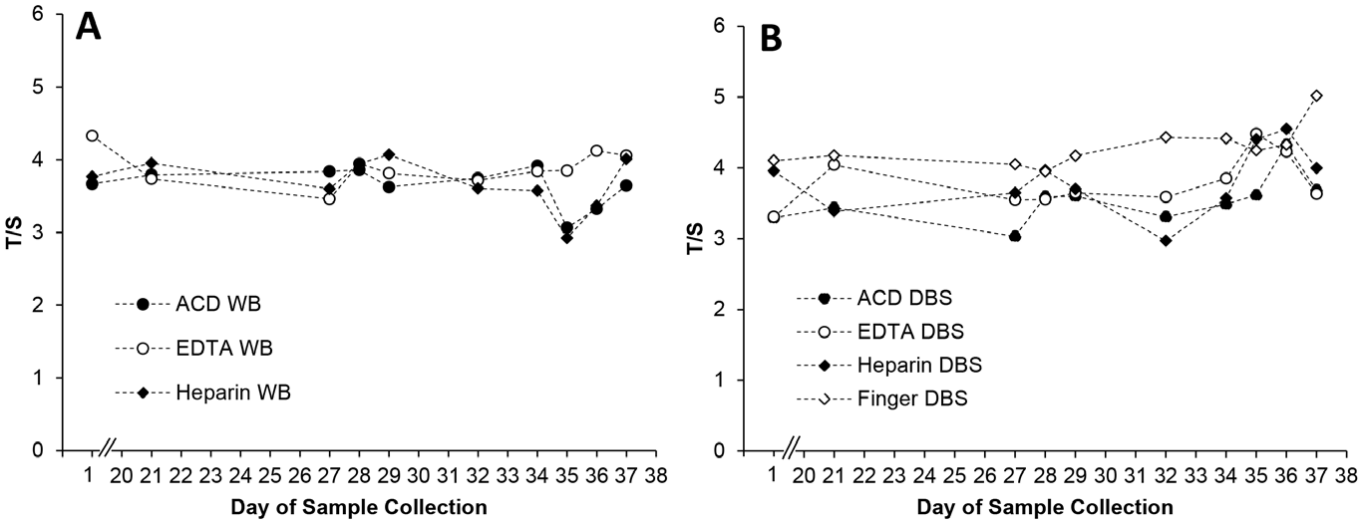
Relative T/S ratio in anti-coagulated whole blood, anticoagulated DBS and finger prick DBS samples over many days. Relative T/S ratios in whole blood collected into three anticoagulants from a single individual on 10 different days (panel A) and DBS created from the anticoagulated whole blood and from finger prick on the same days (panel B) were highly variable.

To determine the day-to-day variability and effect of anticoagulant on T/S ratios, WB obtained by arm venipuncture was collected into EDTA, ACD and heparin tubes, from a single healthy volunteer on 10 different days over a 5-week period. For each type of anticoagulant, WB was either blotted onto a DBS (WB→DBS), or frozen as is (WB). In addition, fingertip blood was spotted directly onto paper DBS (direct finger DBS). Figure 4 (A,B) shows the WB and DBS inter-day T/S variability, respectively, for the three anticoagulants. For WB, the inter-day T/S CV (n = 10) was lowest with EDTA (6.2%) and highest (9.5%) with heparin. Nevertheless, there was no significant difference between the three anticoagulants, whether considering WB (Friedman’s test p = 0.5) or WB→DBS (p = 0.12).However, when the comparison included direct finger DBS, the T/S ratios were significantly different between the groups (p = 0.003). The higher T/S ratio observed for direct finger DBS was responsible for the difference. Inter-day variability was generally higher for WB→DBS (%CV range =  8.9–14.3) compared to WB (6.2–9.5) but there was no significant difference between the two (n = 30, paired t-test, p = 0.79).

### Effect of blood sample storage

#### Blood storage temperature

**Figure 5:**
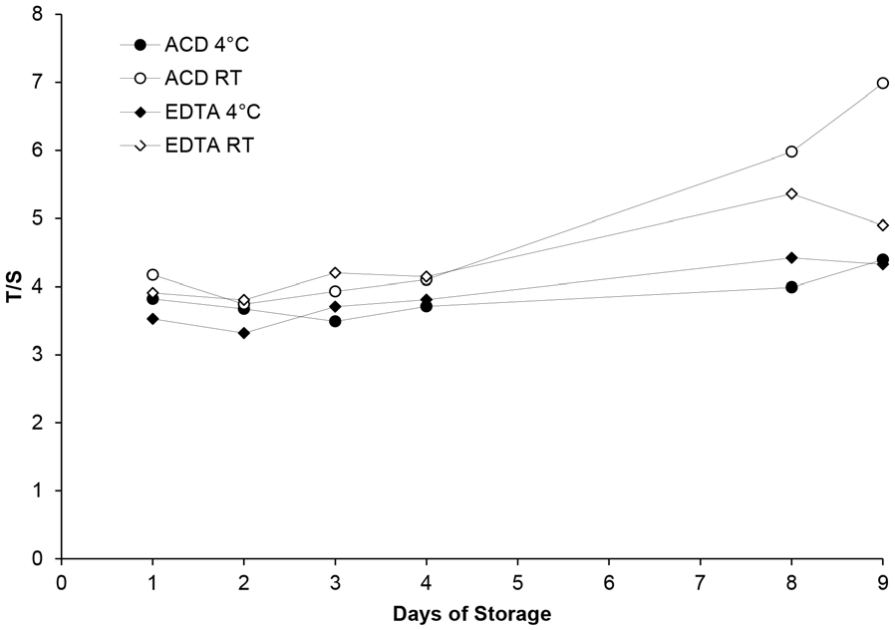
Stability of T/S measurements in anticoagulated whole blood samples in different storage conditions. ACD and EDTA anticoagulated whole blood was stored at 4°C and room temperature (RT) for nine days, and daily T/S ratios are shown.

To test the stability of the T/S measurement, anticoagulated (ACD or EDTA) WB samples were left at either room temperature (RT) or 4°C for 9 days. Given that heparin showed a higher variability, it was not used further. Each day at the same time of day, 100 µL was transferred to a new tube and stored at -80°C. The T/S of WB samples stored at either 4°C or RT differed after 24h, with the greatest change in T/S observed at RT for both anticoagulants (Figure 5). Overall, T/S remained stable for up to 4 days. At 4°C, day 4 ACD-WB and EDTA-WB T/S were increased by 3.8% and 6.0% respectively (n = 1), relative to day 1. For samples stored at RT, similar small increases (4.8% and 4.7%) were observed by day 4 (n = 1). In contrast, storage for 8 days or more resulted in noticeably higher T/S ratios, up to 34% higher after 9 days at RT.

#### Freezing and Thawing

**Figure 6:**
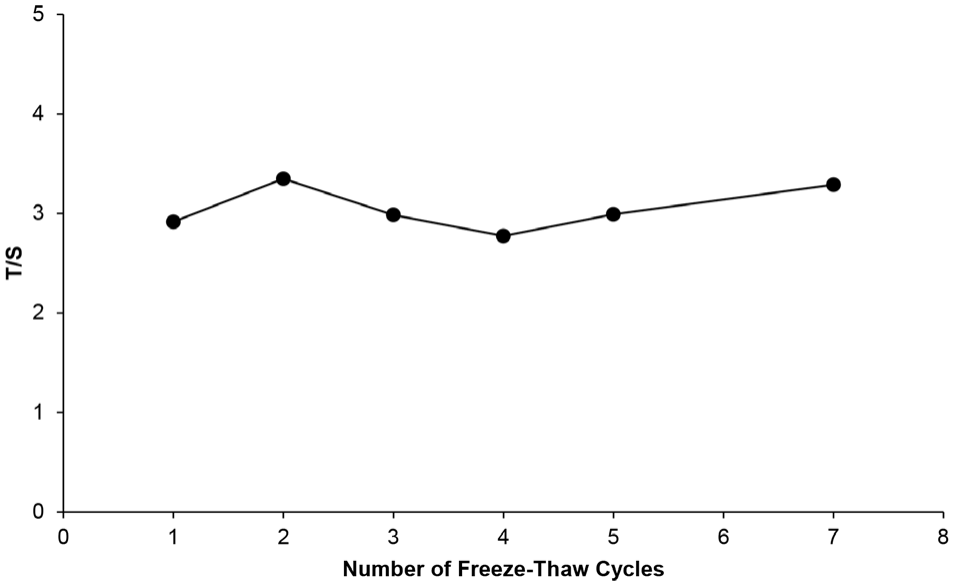
The effect of repeated freezing and thawing on T/S ratio measurements. DNA was extracted from whole blood aliquots obtained at each of seven consecutive freeze-thaw cycles and T/S ratios were determined.

ACD WB was subjected to 7 cycles of freezing at -80°C, and thawing at RT. After each thawing, an aliquot was obtained for immediate DNA extraction. Relative T/S remained stable through freeze-thawing, showing a CV of 7.3% (Figure 6).

### Relative T/S measurements in DBS

As mentioned above, WBDBS T/S was comparable to WB T/S. We investigated what other factors specific to DBS may influence the measurement.

#### Inter-spot Variability

The volume of blood used to blot anticoagulated WB onto paper was constant at 80 µL. However, there was some variation in the amount of blood per spot obtained directly from a finger prick. Some clotting may also occur during finger prick DBS collection. To determine whether this may influence T/S, ACD, EDTA and heparin WB→DBS, as well as direct finger DBS (n = 3 for each type of sample for a total of n = 12) were extracted as per usual using six discs from a single spot, as well as one disc from six distinct sequential spots. No significant difference was seen in the T/S ratios (Mann-Whitney test p = 0.11).

#### Inter-extraction variability

To determine whether the variability observed between WB T/S and that of WB blotted onto DBS (Table 2) may be related to DNA extraction (each type of sample had been extracted in batch), ACD, EDTA and finger DBS samples were randomized and re-extracted. There was no significant difference between the T/S of the original extracts compared to the random extracts (n = 25, 3.87 ± 0.47 *vs.* 3.79 ± 0.31, p = 0.40; Wilcoxon signed-rank).In addition, DBS obtained directly from fingertip once again showed higher T/S ratio.

**Table 2:** Relative T/S ratio (n = 10) measured in WB collected by venipuncture in various anticoagulants and DBS samples (blotted with WB or directly from the fingertip) from a single individual.

	T/S ratio [Mean ± SD (%CV)]			
Anticoagulant	WB	DBS	DBS	P value^a^
	DNA extracted in batches		DNA extracted after randomization	
ACD	3.6 ± 0.3 (7.1)	3.6 ± 0.3 (9.5)	3.5 ± 0.3 (7.9)	0.49
EDTA	3.9 ± 0.2 (6.2)	3.8 ± 0.3 (8.9)	3.8 ± 0.2 (5.2)	0.56
Heparin	3.7 ± 0.3 (9.5)	3.9 ± 0.6 (14.3)	---	0.77
Direct fingertip	---	4.3 ± 0.3 (7.0)^c^	4.0 ± 0.3 (6.3)	---
P value^b^	0.50	0.003	0.005	---

a Wilcoxon signed-rank test to compare between WB and WBDBS of the same anticoagulant

b Friedman’s test to compare anticoagulants within WB and within DBS

c DBS made from finger prick spotted directly onto paper (no anticoagulant)

Abbreviations: T/S  =  relative telomere length ratio, SD  =  standard deviation, CV  =  coefficient of variation, WB  =  whole blood, DBS  =  dried blood spot, ACD  =  acid citrate dextrose anticoagulated, EDTA  =  Ethylenediaminetetraacetic acid anticoagulated

### T/S in DBS vs. PBMC vs. anticoagulated whole blood samples

**Figure 7:**
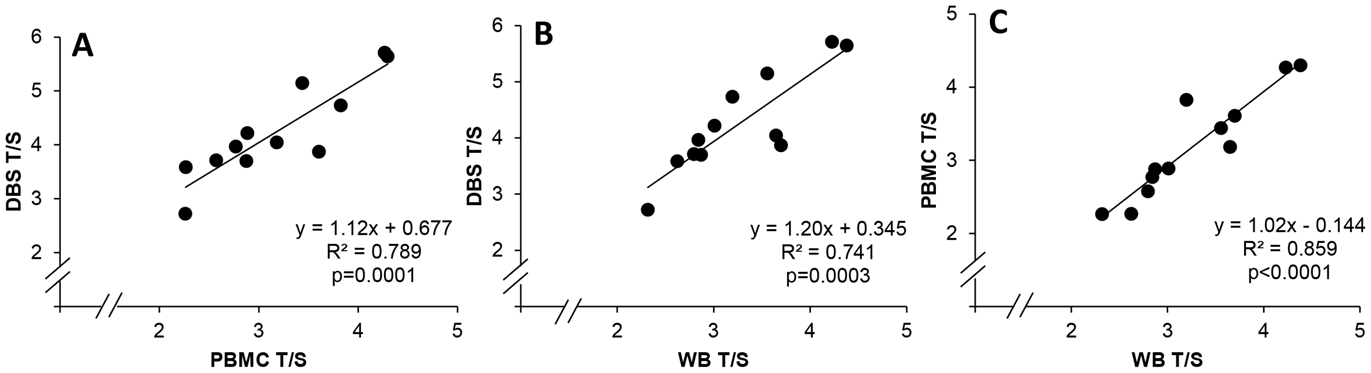
Comparison of T/S measurements in DBS, PBMC and WB samples. T/S ratios were determined for DNA from DBS (n = 12), PBMC (n = 12) and WB (n = 12) samples made from a single blood draw from 12 distinct individuals. Pearson’s R**^2^** and p values are shown.

To determine the influence of blood cell processing on T/S ratios, peripheral blood was collected from 12 healthy volunteers, using both ACD and EDTA tubes, during the same venipuncture . PBMC were prepared from the ACD blood and their T/S determined. T/S was also determined for EDTA WB, as well as for DBS prepared from the EDTA WB. Without exception, all T/S ratios from DBS were higher than those from both PBMC and WB (p<0.001, paired t tests). The correlations between each type of blood sample are shown in Figure 7 (A–C). PBMC and WB had the highest correlation between them.

### Effect of site of blood draw

**Figure 8:**
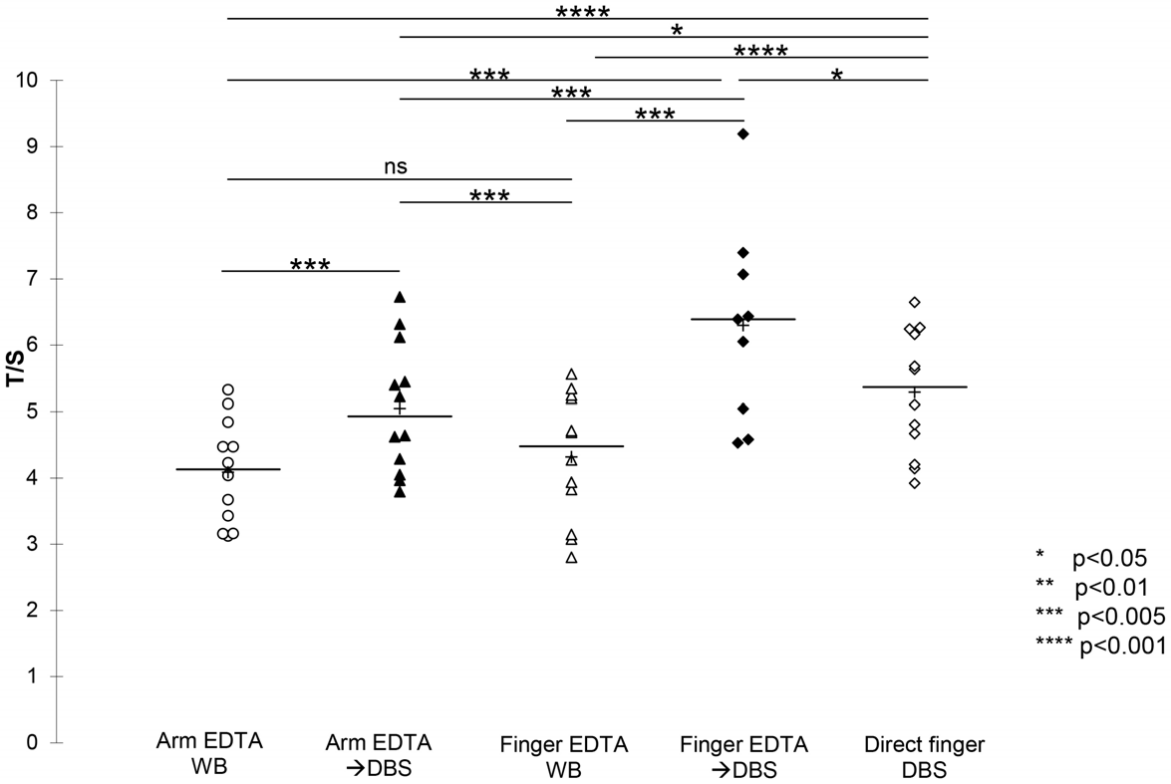
The effect of site of blood draw on T/S ratio measurements. T/S ratios were measured for DNA from arm EDTA whole blood, arm whole bloodDBS, finger EDTA WB, finger EDTADBS and direct finger prick blood made from a single blood draw from 12 distinct individuals. P values are shown, without adjustment for multiple comparisons. ns  =  not significant (p>0.05). The line represents the median and the + represents mean.

To investigate further the differences observed between T/S measurements of WB and DBS obtained from a single subject, 12 volunteers aged 23–50 years old were studied. For each person, blood was drawn from arm venipuncture into EDTA (arm EDTA WB) and part of it was blotted onto paper (arm EDTA WBDBS). In addition, blood was collected from a finger prick into a 500 µL pediatric EDTA tube (finger EDTA WB) and part of it was blotted onto paper (finger EDTA→DBS), and finally, finger prick blood was spotted directly onto paper (direct finger DBS).Finger EDTA blood was spotted onto DBS for 9/12 volunteers due to insufficient blood remaining. The mean ± SD and ranges of the T/S ratios are shown in Table S1.There were significant differences in T/S when comparing the five groups by Friedman’s test (p<0.0001). Pair wise comparisons (Wilcoxon) are shown in Table S2 and Figure 8. All pairs were significantly different (p<0.021) except for venous EDTA WB *vs.* finger EDTA WB, although no adjustments were made for multiple comparisons.

## Discussion

Comparing T/S ratios between laboratories can be challenging. Differences may arise from different qPCR instruments, single copy nuclear genes used and standard calibration, and ultimately, assay variability. Our monoplex assay CV, calculated based on more than 38 independent runs over a period of several months varied from 5% to 17%, depending on the DNA concentration and T/S ratio of the internal controls, suggesting that a single internal control might not fully reflect assay variability. Other groups have reported CV values of 5 to 8% [11], [17], [18], [19].T/S can be measured by qPCR from a variety of blood sample types, including WB, DBS and PBMCs. Our results showed a high correlation between these three types of blood samples, although DBS consistently yielded higher T/S values.

As our experiments were carried out in early 2009, monoplex qPCR was used for most investigations presented herein. We later confirmed a strong correlation between TL measured by monoplex and multiplex qPCR, such that the method itself would be unlikely to affect the observations made here. As multiplex qPCR is time saving, reduces reagent costs, and can reduce assay variability, future studies, especially high throughput ones would benefit from using the multiplex assay. Of note, we observed higher variability on the plate’s edges with the multiplex assay, particularly in the plate corners, something that was not seen with the monoplex assay (data not shown).

We conducted a detailed analysis of potential factors that can influence T/S measurements. Among the factors that did not appear to play an important role were the type of anticoagulant used during whole blood collection, freeze-thawing of study samples, and the temperature ramping speed during qPCR. Blood collected in heparin appeared to show higher TL variability but it did not significantly differ from that collected in EDTA or ACD.

Storage can affect the quality of the samples and determines whether their future use is possible. It has been reported that the quality of DNA is not adversely affected by WB storage at 4°C or RT for up to 24h before processing [20], [21]. Cell viability is reportedly decreased after 48h resulting in the degradation of nucleic acids, such that it is usually recommended that WB samples be stored at 4°C and processed within 24h of collection [22]. However, in a more recent study, the quality of genomic DNA obtained from WB and DBS was not influenced by storage at RT for 12 days [23]. Our results showed that WB TL measurements were stable for up to 4 days at both temperatures. However, storage for longer periods had a significant impact on the measured T/S ratios, and suggested faster degradation at RT, whereby T/S ratio increased as time went by. A possible explanation could be that quadruplex structures at the 3’ end of telomeric DNA [24] become more accessible to PCR polymerase over time at RT, resulting in a larger T value, while the S gene remains unchanged. Long-term freezing of whole blood, and repeated freezing and thawing are not recommended[25]but we found no evidence that repeated freeze-thawing affected WB TL measurements.

DBS are a non-invasive, inexpensive, and widely used method of blood sample collection. They are suitable for genetic, biomarker, and analyte tests typically done using whole blood [26]. For both WB and DBS, the low inter-day T/S variability observed suggests that both are suitable for T/S measurements, thus allowing greater flexibility in sample collection for research purposes. However, several factors significantly affected WB and DBS T/S measurements. Among them, the site of blood draw, and the blotting of the WB onto paper to prepare a DBS. Indeed, the observed differences in T/S ratios between finger-DBS collected directly from a finger prick onto paper, anti-coagulated WB collected from arm venipuncture, and DBS derived from anti-coagulated arm venipuncture WB blotted onto paper strongly suggest that blood collection methods affect TL measurements. More specifically, we found that the action of blotting anti-coagulated WB onto paper, whether collected from the arm or the finger, resulted in a significant increase in T/S measures. Furthermore, we initially noted that finger prick blood collection directly onto paper yielded significantly higher TL than that seen in the arm venous blood from the same individual. We initially hypothesized that this may be due to the large difference in blood vessel diameter between arm veins and finger capillaries. One limitation to using mixed cell samples such as WB, PBMCs, or DBS for research purposes is that little is known regarding the relative contribution of various cell subtypes to the overall T/S. Finger capillary blood has different large leukocyte (granulocyte) counts than arm venous blood [27], [28], [29], [30], [31], and granulocytes have longer TL than lymphocytes [32]. This could have explained our observation of a higher T/S ratio in finger prick DBS. However, finger prick blood collected into EDTA showed similar T/S as arm EDTA WB. Since it was only upon blotting of that finger prick EDTA WB onto paper that the increase in T/S was observed, we conclude that the paper or the absence of anti-coagulant prior to blotting may be responsible for the observed increase in T/S. We could not resolve this question. It is possible that using paper specially designed for use at RT would prevent this effect if it relates to DNA degradation. Anecdotally, we observed large differences in DBS T/S between a paper designed for storage at room temperature and another not intended for room temperature use, after many years of storage (data not shown).

In conclusion, the realization that TL influences biological functions ranging from aging to carcinogenesis has highlighted the need for techniques and tools that can provide accurate information on TL data. To realize fully the potential of TL research using the inexpensive and high throughput qPCR method, it is essential to understand what factors may influence this measure. WB should not be stored for more than 4 days before DNA extraction. Although DBS are convenient and can be used reliably to measure TL, one should exercise caution when gathering and comparing such data as paper-blotted blood (DBS) clearly yield higher T/S values than other types of samples such as WB or PBMC. Site of blood collection (arm vs. fingertip) does not affect T/S as long as both are collected into anticoagulant and treated similarly.

## Supporting Information

Table S1 **T/S ratios for the 12 volunteers aged 23 to 50 years for blood collected from different sites with or without blotting onto paper.**
(DOCX)

Table S2 **Pairwise comparison of blood draw sites including p value (Wilcoxon) and common elements.**
(DOCX)
