# Supplementary material for: Blood and Dried Blood Spot Telomere Length Measurement by qPCR: Assay Considerations
Source: PLoS One. 2013 Feb 25;8(2):e57787. doi: 10.1371/journal.pone.0057787 (PMC3581490; doi:10.1371/journal.pone.0057787)
Supplement: Table S1 — T/S ratios for the 12 volunteers aged 23 to 50 years for blood collected from different sites with or without blotting onto paper. (DOCX) [file pone.0057787.s001.docx]

Table S1. T/S ratios for the 12 volunteers aged 23 to 50 years for blood collected from different sites with or without blotting onto paper.

|  | T/S ratio (n=12) | |
| --- | --- | --- |
| Variable | Mean ± SD | Range |
| Arm EDTA WB | 4.09 ± 0.78 | 3.16 – 5.32 |
| Arm EDTA WB🡪DBS | 5.05 ± 0.98 | 3.79 – 6.73 |
| Finger EDTA WB | 4.32 ± 0.96 | 2.80 – 5.34 |
| Finger EDTA WB🡪DBS | 6.30 ± 1.5 | 4.53 – 7.39 |
| Finger DBS | 5.29 ± 0.94 | 3.92 – 6.65 |
| P value^a^ | p<0.0001 | --- |

^a^Friedman’s test to compare T/S within the five groups

Abbreviations: T/S = relative telomere length ratio, SD = standard deviation, EDTA = Ethylenediaminetetraacetic acid anticoagulated, WB = whole blood, DBS = dried blood spot
