# Supplementary material for: Blood and Dried Blood Spot Telomere Length Measurement by qPCR: Assay Considerations
Source: PLoS One. 2013 Feb 25;8(2):e57787. doi: 10.1371/journal.pone.0057787 (PMC3581490; doi:10.1371/journal.pone.0057787)
Supplement: Table S2 — Pairwise comparison of blood draw sites including p value (Wilcoxon) and common elements. (DOCX) [file pone.0057787.s002.docx]

Table S2. Pairwise comparison of blood draw sites including p value (Wilcoxon) and common elements.

| Pair # | Variables (in pairs) | P value | Common elements |
| --- | --- | --- | --- |
| 1 | arm EDTA WB & arm EDTA DBS | 0.001 | Site, EDTA |
| 2 | arm EDTA WB & finger EDTA WB | 0.47 | EDTA, WB |
| 3 | arm EDTA WB & finger EDTA DBS | 0.004 | EDTA |
| 4 | arm EDTA WB & direct finger DBS | 0.0005 | --- |
| 5 | arm EDTA DBS & finger EDTA WB | 0.001 | EDTA |
| 6 | arm EDTA DBS & finger EDTA DBS | 0.004 | EDTA, DBS |
| 7 | arm EDTA DBS & direct finger DBS | 0.021 | DBS |
| 8 | Finger EDTA WB & finger EDTA DBS | 0.004 | site, EDTA |
| 9 | finger EDTA WB & direct finger DBS | 0.0005 | site |
| 10 | finger EDTA DBS & direct finger DBS | 0.020 | Site, DBS |

Abbreviations: EDTA = Ethylenediaminetetraacetic acid anticoagulated, WB = whole blood, DBS = dried blood spot
